# Longevity

**Published:** 1881-11

**Authors:** 


### LONGEVITY.

M. de Solaville contributes to the *Revue Scientifique* a valuable article on this subject, in which he brings together some of the most recent data. He analyzes the results of recent European censuses by ages, and the register of deaths also by ages. If we strike a mean of the census from 1869 to 1872 we find that Europe (exclusive of Russia, Turkey, and some small Southern States) possessed in 1870 a mean population of 242,940,376, classed as follows from the point of view of advanced ages:—17,313,715 of more than 60 years, 79,859 of more than 90, and 3,108 of more than 100 years; *i.e.*, 1 inhabitant in 12 of more than 60, 1 in 2,659 of more than 90, and 1 in 62,503 of more than 100. Women, M. Solaville finds, are more numerous in extreme old age than men, and the difference increases with the age. Thus at 60 years the advantage is with the women in the proportion of 7 per cent.; at 90 and above it rises to 45, and with centenarians to 60 per 100. It is in France that we find the greatest relative number of inhabitants at the age of 60 and upwards; but it is not so for centenarians, of which France has less than all the other States of Europe except Belgium, Denmark, and Switzerland. From a calculation of deaths by ages the result is reached that, to the total deaths, those at the age of 90 and upward bore the following proportions to the countries named, and arranged according to the decreasing order of importance; Great Britain,9.73; Sweden,7.39; France,6.58; Belgium,6.07; Switzerland,6.00; Holland, 4.47; Italy, 3.76; Bavaria, 3.42; Prussia, 3.06; Austria, 2.61. The result is in accordance with what we know of the mean age of the deceased in the same countries.

As to whether great longevity is increasing or diminishing for the same number of inhabitants, our data refers only to France.

If we take two periods sufficiently distant from each other to allow a change of any importance to be produced, we find, in the 14 years of the period 1824–37, a mean annual number of deaths among centenarians of 152, or 1 to 217,105 inhabitants. In the eight years of the period 1853–60, we only find a mean annual number of 11 centenarian deaths in a population which has increased 20 per cent. But if great ages appear to have diminished the mean life has very sensibly increased, a result much more favorable. A certain number of centenarians have made known their regimen. Notwithstanding some very rare examples to the contrary we must place in the first rank temperance, sobriety, and regular habits; then come heredity, relative comfort, the absence of strong and pregnant emotion, as far as possible a country life, and finally a healthy and quiet calling.—*N. Y. Medical Times.*

